# Predicting dispensing errors in community pharmacies: An application of the Systematic Human Error Reduction and Prediction Approach (SHERPA)

**DOI:** 10.1371/journal.pone.0261672

**Published:** 2022-01-04

**Authors:** Ahmed Ashour, Denham L. Phipps, Darren M. Ashcroft

**Affiliations:** 1 Division of Pharmacy and Optometry, Centre for Pharmacoepidemiology and Drug Safety, School of Health Sciences, Faculty of Biology, Medicine and Health, University of Manchester, Manchester, United Kingdom; 2 NIHR Greater Manchester Patient Safety Translational Research Centre, Manchester Academic Health Science Centre, University of Manchester, Manchester, United Kingdom; 3 NIHR School of Primary Care Research, Manchester Academic Health Science Centre, University of Manchester, Manchester, United Kingdom; The University of Sydney School of Pharmacy, AUSTRALIA

## Abstract

**Introduction:**

The objective of this study was to use a prospective error analysis method to examine the process of dispensing medication in community pharmacy settings and identify remedial solutions to avoid potential errors, categorising them as strong, intermediate, or weak based on an established patient safety action hierarchy tool.

**Method:**

Focus group discussions and non-participant observations were undertaken to develop a Hierarchical Task Analysis (HTA), and subsequent focus group discussions applied the Systematic Human Error Reduction and Prediction Approach (SHERPA) focusing on the task of dispensing medication in community pharmacies. Remedial measures identified through the SHERPA analysis were then categorised as strong, intermediate, or weak based on the Veteran Affairs National Centre for Patient Safety action hierarchy. Non-participant observations were conducted at 3 pharmacies, totalling 12 hours, based in England. Additionally, 7 community pharmacists, with experience ranging from 8 to 38 years, participated in a total of 4 focus groups, each lasting between 57 to 85 minutes, with one focus group discussing the HTA and three applying SHERPA. A HTA was produced consisting of 10 sub-tasks, with further levels of sub-tasks within each of them.

**Results:**

Overall, 88 potential errors were identified, with a total of 35 remedial solutions proposed to avoid these errors in practice. Sixteen (46%) of these remedial measures were categorised as weak, 14 (40%) as intermediate and 5 (14%) as strong according to the Veteran Affairs National Centre for Patient Safety action hierarchy. Sub-tasks with the most potential errors were identified, which included ‘producing medication labels’ and ‘final checking of medicines’. The most common type of error determined from the SHERPA analysis related to omitting a check during the dispensing process which accounted for 19 potential errors.

**Discussion:**

This work applies both HTA and SHERPA for the first time to the task of dispensing medication in community pharmacies, detailing the complexity of the task and highlighting potential errors and remedial measures specific to this task. Future research should examine the effectiveness of the proposed remedial solutions to improve patient safety.

## 1. Introduction

Reducing preventable harm in healthcare, and the errors that lead to them, has long been recognised as a patient safety priority, with recent reports estimating that 5% of patients are exposed to preventable harm during their medical care [1]. In England, an estimated 11 million medication errors of clinical significance occur annually [2], and in 2017, the World Health Organization (WHO) set a global patient safety challenge to reduce the overall burden of medication-related harm by 50% within five years [3, 4]. Community pharmacies will play an important role in achieving this challenge as the number of prescriptions dispensed by pharmacies increases across the world, with data reporting that the number of items dispensed in England reaching 1.01 billion items in 2019, an increase of 30% over 10 years [5].

There are several ways that errors can occur within a community pharmacy setting. Community pharmacists are tasked with screening prescriptions requested from other healthcare professionals for patients; however, this screening sometimes fails to identify medicines that are unsuitable for a patient’s condition or characteristics, resulting in medicines that are dispensed correctly against the prescription, but unsafe for the patient. The second type of errors that community pharmacists must identify and rectify are dispensing errors, where medicines are dispensed that are not identical to the orders of the prescriber, or incorrect instructions are printed on the labels attached to the medicines. These types of errors can also be missed by the pharmacist, or pharmacy support staff, during accuracy checks of dispensed medicines. Medication errors occurring at the point of dispensing in community pharmacies have been the focus of multiple studies, utilising a range of retrospective methods are well established within the literature [6–8]. However, studies have suggested that applying prospective risk analysis methods can identify different risks than those discovered through retrospective analysis of incidents [9]. In order to complement the studies available retrospectively analysing incidents within community pharmacy, various prospective risk analysis methods should be applied to the complex task of dispensing medicines [10].

Prospective risk analysis methods have a proven track record of improving outcomes in high-risk industries (e.g. crane operations) [11], as well as identify key insights within healthcare processes [12, 13]. These methods are particularly beneficial for their ability to raise awareness of risks, as well as their usefulness in areas that may not have comprehensive incident reporting systems [9]. Various prospective risk analysis methods exist, and they tend to follow systematic instructions that focus on accurately describing the task at hand, as well as identifying the related issues that could pose significant or crucial safety consequences. Two of the most common methods are the Failure Mode and Effects Analysis (FMEA) and the Systematic Human Error Reduction and Prediction Approach (SHERPA). While both risk analysis methods have similar steps, significant differences rely on the approach used to identify the risks. FMEA relies on creative thinking and experienced subject matter experts to firstly describe the stages of a task before identifying potential failure modes at each stage and assigning causes, effects, and corrective actions to each failure mode [14]. On the other hand, SHERPA relies on the availability of a Hierarchical Task Analysis (HTA), which decomposes a task into relevant sub-tasks and aims to accurately describe the actions required to be taken to successfully achieve a goal, coupled with an error taxonomy which is applied by subject matter experts to describe relevant potential errors, before remedial measures are proposed [15, 16]. While both these tools have similar objectives to analyse risk, SHERPA offers a valuable insight in predicting potential errors that can supplement findings from FMEA [17], due to its more granular approach of being grounded in task analysis.

To date, limited investigations have applied prospective risk analysis methods in a community pharmacy setting, and particularly to the task of dispensing medicines. A literature review conducted in 2017 by Stojkovic et al. investigating the use of prospective risk analysis tools in a pharmacy did not identify any studies applying either FMEA or SHERPA to community pharmacy dispensing [18]. Since then, FMEA has been applied twice in a community pharmacy setting: once in a German community pharmacy; and the second in a Serbian community pharmacy [19, 20]. Both of these studies have shown the insights to be generated by applying a prospective risk analysis method in a pharmacy setting. These studies individually identified 30 [20] and 39 [19] failure modes, with many overlapping between the two, based on focus group discussions and brainstorming sessions. The majority of the identified failure modes were omissions of checks or actions by the pharmacist, or pharmacy support staff, with consequences that could lead to significant patient harm.

Additionally, a key stage in the prospective risk analysis methods is the generation of remedial measures to avoid errors occurring. These suggestions can be categorised as weak, intermediate or strong, based on a patient safety action hierarchy developed by the US Veteran Affairs National Centre for Patient Safety [21], which ranks strongest interventions as those relying least on human behaviour, as recommended by Human Factors and Ergonomics (HFE) principles [20]. The objective of this study was to use a prospective error analysis method to examine the process of dispensing medication in a community pharmacy setting and identify remedial solutions to avoid potential errors, categorising them as strong, intermediate, or weak based on the aforementioned patient safety action hierarchy.

## 2. Method

This study used a qualitative design, that was influenced by a realist approach, with data collected through focus group discussions and non-participant observations of subject matter experts (practising community pharmacists) to develop the HTAs, and a focus group formed subsequently to apply SHERPA to the developed HTA, to identify the potential errors that exist in the dispensing task. This is in line with the realist approach to qualitative research, which aims to uncover the reality of a phenomenon, separate to its interpretation or perception.

### 2.1. Participants

Participants were recruited on a convenience basis from a community pharmacist research user group at the authors’ institution. The group comprised of 7 experienced community pharmacists who meet regularly to discuss pharmacist and academic-led patient safety initiatives and provide crucial insights to researchers on the feasibility of patient safety projects. Prior to this focus group, the authors had engaged with this community pharmacists research user group on other research projects previously. The participants’ community pharmacy experience ranged from 8 years to 38 years, with a mean experience of 17.2 years (SD = 10.3).

### 2.2. Producing the HTA

Critical to applying SHERPA, is the presence of a HTA of the task under investigation. In this study the HTA was produced through data collected from both non-participant observations, and focus group discussions, based on data collection methods applied by Raduma-Tomas et al., to develop a HTA for doctors’ handovers in acute medical assessment units [22] and Ashour et al., to develop a similar HTA for dispensing in community pharmacies [23]. Non-participant observations at the places of work of three of the focus group participants, altogether observing pharmacist and pharmacy staff within the pharmacy for 12 hours. Observations were conducted by the first author, an experienced community pharmacist familiar with the task of dispensing medicines. The observer gathered free-hand notes of the sub-tasks conducted to dispense a prescription in the pharmacy. Each set of notes were written up into case studies upon completion of the observation, which formed the text which was analysed to identify the sub-tasks and plans to generate the HTA. The HTA was produced based on the steps described by Annett [24], and reported by Phipps et al., [25].

This HTA was then presented to the participants of the focus group, and they were asked to discuss variations and inaccuracies in the HTA, with discrepancies discussed between the pharmacists until consensus was achieved that the HTA was representative of a typical dispensing process within a community pharmacy.

### 2.3. Applying SHERPA

Stanton et al. [26] described the method for conducting a SHERPA analysis, which has been summarised below. Altogether three focus groups were conducted, each lasting between 57 to 85 minutes. All three focus groups included the same seven participants. Prior to the beginning of the first focus group, participants were given a training session on how to conduct a SHERPA analysis, as described by Stanton et al. [26]:

1. Categorise each step of the dispensing task as one of the following: action; retrieval; checking; selection; information; or communication
2. Apply the relevant potential errors from the pre-set error taxonomy (shown in Table 1)
3. Describe the consequences of the potential error if it occurred
4. State whether there are any potential steps to recover from the potential error
5. Rate the chances of the error occurring in terms of low (L; hardly occurs), medium (M; occasionally occurs); or high (H; frequently occurs)
6. Determine the criticality if the potential error occurred in terms of low (L; barely noticeable effect), medium (M; a potentially noticeable but transient effect), or high (H; a potentially life-threatening)
7. Propose potential remedial measures that could prevent the error from occurring

**Table 1:** Error taxonomy descriptions.

Class of behaviour	Task Category Error
Action	A1. Too long or too short
	A2. Mistimed
	A3. Wrong direction
	A4. Too little/too much
	A5. Misaligned
	A6. Wrong object
	A7. Wrong action
	A8. Omitted
	A9. Incomplete
	A10. Wrong action and wrong object
Check	C1. Omitted
	C2. Incomplete
	C3. Wrong object
	C4. Wrong check
	C5. Mistimed
	C6. Wrong check, wrong object
Retrieval	R1. Information not obtained
	R2. Wrong information obtained
	R3. Information retrieval incomplete
Communication	I1. Information not communicated
	I2. Wrong information communicated
	I3. Information communication incomplete
Selection	S1. Omitted
	S2. Wrong selection made

Participants were then given copies of the HTA, as well as templates that were pre-populated with the lowest level of sub-task from the HTA on dispensing, alongside columns representing each step of the SHERPA process. The focus groups were facilitated by the first author, who has experience as a community pharmacist, and the second author, a human factors expert with experience researching dispensing in community pharmacies. Sub-tasks were then discussed in numerical order, with participants then asked to discuss each column in turn. Once the participants were familiar with sub-task being considered, they categorised the sub-task based on the type of behaviour detailed in Table 1. Then, each category error was applied to understand whether or not it could be a potential error in practice. Each category error example was then described, as well as identifying any potential recovery steps that were present later in the task, before participants collectively rated the chances of the error occurring and the criticality of the error if it did occur. Finally, participants were asked to list potential remedial measures that could prevent the error from happening.

Following the completion of the focus groups, the proposed remedial solutions were categorised according to the Veteran Affairs National Centre for Patient Safety action hierarchy [21], by the first author, with categorisations checked and confirmed by the second author.

### 2.4. Ethics

Ethical approval was granted by the [BLINDED FOR REVIEW] (2018-4564-6564). All participants gave informed written consent to participate in the study.

## 3. Results

### 3.1. Hierarchical task analysis

**Fig 1:**
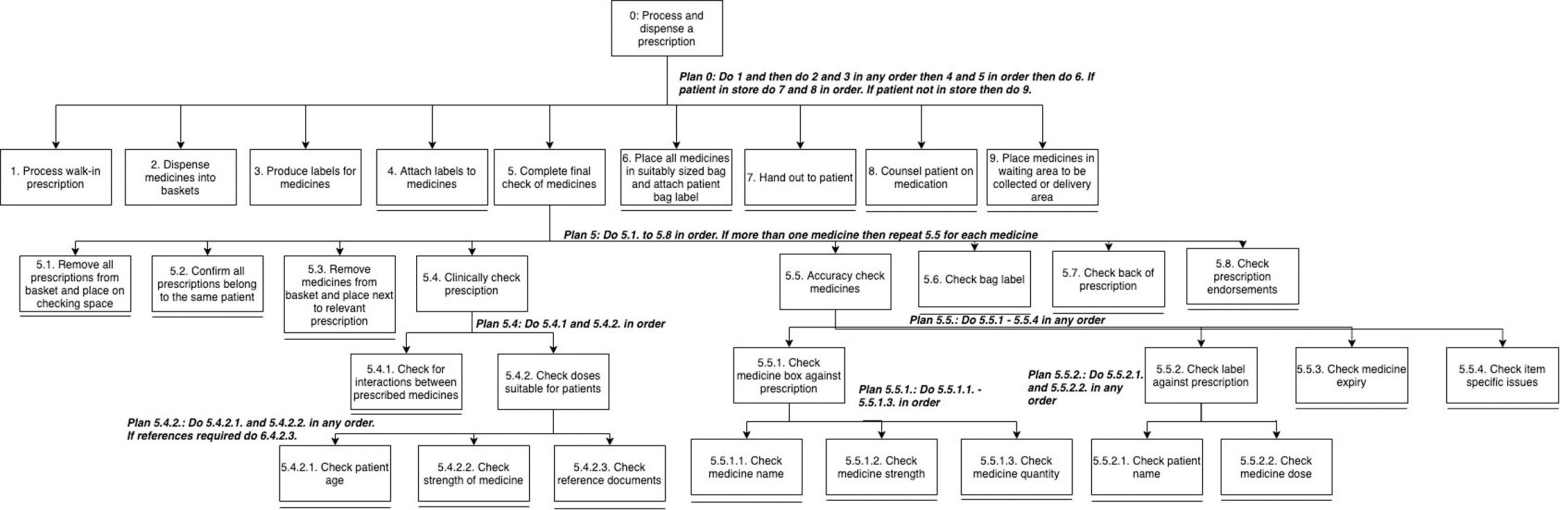
HTA of dispensing task including complete high-level subtasks and expanded view of sub-task 3.0. A single black line beneath a sub-task indicates it’s the lowest level.

A HTA was produced for the dispensing task, which consisted of 9 sub-tasks, ranging from physical to cognitive steps, and varying levels of complexities. Fig 1 presents the high-level sub-tasks, with sub-task 3 expanded with lower sub-tasks presented.

### 3.2. Potential errors

In total 88 potential errors were identified for the dispensing task, 28 of them involving task step 3 (produce labels for prescription) and a further 28 in task step 5 (complete final check of medicines). Of these errors, 42 (47.7%) were related to actions and a further 29 errors (33%) were related to checks. The potential errors, and their corresponding task categories, are presented in Table 2. An excerpt of the SHERPA analysis is presented in Table 3, showing the potential errors, and suggested remedial measures for task step 5, which covers the accuracy and clinical check of the medicines dispensed by the pharmacist. Table 3 shows the individual columns representing the task, the potential error code, and its consequence, in addition to the likelihood and criticality of it happening. In the last column, potential remedial measures are presented.

**Table 2:** The number of identified potential errors and corresponding task category errors.

Category	Error mode	Number of potential errors
Action	A6—Wrong object	14
	A7—Wrong action	2
	A8—Omitted	15
	A9—Incomplete	11
Check	C1—Omitted	19
	C2—Incomplete	10
Retrieval	R1—Information not obtained	1
	R2—Wrong information obtained	8
Communication	I1—Information not communicated	2
	I2—Wrong information communicated	1
	I3—Information communication incomplete	1
Selection	S1—Omitted	1
	S2—Wrong selection made	3
		Total: 88

**Table 3:** Excerpt of completed SHERPA table detailing output for sub-tasks 5.1 to 5.6.

Task step	Type of task step	Error Code	Description	Consequence	Recovery	Probability of Error Occurring	Criticality of Error if Occurred	Remedial measures
5.1	Action	A8	Prescriptions not removed from basket	No thorough checking of all prescriptions		H	L	Dedicated clear area for checking the prescription
								Operating procedure on how to organise medicines once dispensed in a basket
								Clearly marked number of prescriptions on each prescription (i.e., 1 of X)
								Anthropometrically appropriate desk for the pharmacist
								Consideration for colour contrasting (e.g., avoiding a white prescription in white basket on a white desk)
		A9	Not all prescriptions removed from basket	No thorough checking of all prescriptions		H	L	Ensure correct size of baskets available and used depending on number of dispensed items
5.2	Check	C1	No confirmation that all prescriptions belong to same patient	Medicines for more than one patient combined in one bag	5.6	L	M	Clear checking area with enough space to thoroughly check prescription
		C2	Incomplete confirmation that all prescriptions belong to same patient	Medicines for more than one patient combined in one bag	5.6	L	M	Reduced distractions and interruptions while dispensing
5.3	Action	A6	Incorrect medicines are removed from basket	No thorough checking of all medicines		M	L	Operating procedure on importance of being thorough when checking medicines
		A8	Medicines not removed from basket and placed next to relevant prescription	No thorough checking of all medicines		M	L	Clear checking area with ergonomically informed decisions on equipment used
		A9	Not all medicines are removed from basket and placed next to relevant prescription	No thorough checking of all medicines		M	L	
5.4.1	Check	C1	No check for interactions between medicines	Interaction between medicines can have varied severity		L	L	Importance of clinical checks by pharmacist when checking prescription (e.g., posters on wall)
								Think about splitting the tasks of clinical and accuracy check
								Incorporate technology to aid the task (i.e., check at the computer)
								Forced break/task-switching policy to reduce possibility of unfocused checking
		C2	Incomplete check for interactions between medicines	Interaction between medicines can have varied severity		L	L	Greater awareness of abilities and importance of task switching when unfocused
5.4.2.1	Check	C1	No check for patient’s age	Unsuitable medicine for age		L	M	Introduce sticker for prescriptions belonging to young children to ensure their prescriptions are highlighted
		C2	Incomplete check for patient’s age	Unsuitable medicine for age		L	M	
5.4.2.2	Check	C1	No check for suitability of strength of medicine	Unsuitable medicine for age		L	M	Introduce sticker for prescriptions belonging to young children to ensure their prescriptions are highlighted
		C2	Incomplete check for suitability of strength of medicine	Unsuitable medicine for age		L	M	
5.4.2.3	Check	C1	No referring to reference material	Unsuitable medicine for age		L	M	Ensure reference material on site and to hand during checking task
		C2	Insufficient referring to reference material	Unsuitable medicine for age		L	M	
5.5.1.1	Check	C1	Failure to check medicine name	Wrong medicine dispensed		L	H	Clear area for thorough checking of prescription
								Explore ergonomic issues (e.g., font, size, tall-man lettering)
		C2	Failure to completely check medicines name	Wrong medicine dispensed		L	H	Utilise bar code scanning to check medicines name
5.5.1.2	Check	C1	Failure to check medicine strength	Wrong medicine dispensed		L	M-H	Clear area for thorough checking of prescription
								Standardised strength format (e.g., percentages or strength)—same as prescription
								Introduce colour coding as a supplementary cue for different strengths
		C2	Failure to completely check medicines strength	Wrong medicine dispensed		L	M-H	Isolate high-risk drugs with high potential for error (e.g., Methotrexate 2.5mg or 10mg)
5.5.1.3	Check	C1	Failure to check medicine quantity	Wrong amount of medicine dispensed		L	L	Clear area for thorough checking of prescription
		C2	Failure to completely check medicine quantity	Wrong amount of medicine dispensed		M	L	Ensure pack sizes are consistent (e.g., Clopidogrel 28 and 30 tablets)
5.5.2.1	Check	C1	Failure to check patient name compared to label	Wrong patient’s medicine dispensed		L	L	Clear area for thorough checking of prescription
								Clear up patient profile names
5.5.2.2	Check	C1	Failure to check medicine dose compared to prescription	Wrong dose printed on label		L	M	Clear area for thorough checking of prescription
								Standardised dosing names
5.5.3	Check	C1	Failure to check medicine expiry date	Expired medicines dispensed		M	L	Clear process for checking medicines
								Regular data checking process
								Mark medicines that are expiring within 6 months
								Procedure for checking medicine expiry dates on receipt of stock into the pharmacy
								Ensure expiry dates written on split pack box
								Ensure date opened documented on medicine
								Use bar code scanning to ensure medicines in date
5.5.4	Check	C1	Failure to check medicine related issues	Item related medicine issue failure		M	M	Importance for training on specific items
								Sticker for fridge and CD medicines
		C2	Failure to completely check medicine related issues	Item related medicines issue failure		M	M	Importance for training on specific items
5.6	Check	C1	Failure to check bag label	Wrong bag label on bag		M	H	Clear organised area
								Stick bag label on basket
								Introduce record of dispensing register
								Ensure clear audit trail of who dispensed, accuracy checked, clinically checked, and handed out the prescription

P = Probability of Error Occurring, C = Criticality of Error if Occurred, H = High, M = Medium, L = Low. Refer to Table 2 for error code descriptions.

Omitting a check (C1) was the most likely error to occur, accounting for 19 (21.5%) of the suggested errors. Second was omitting an action (A8) which accounted for 15 (17%) of the errors. Examples of action omissions included failing to affix a label to medication and failing to enter the quantity of medicine to be supplied (when the medication order could not be completely fulfilled in a single dispensing transaction). The focus group discussions identified the main reason for omitting an action to be the pharmacist being distracted or interrupted. Sources of distraction included a telephone ringing or another staff member making a request of the pharmacist during the dispensing process, which lead to the pharmacist switching tasks and possibly forgetting his or her place in the original task when returning to it.

The errors considered to occur less frequently were those relating to communication or selection of medicines. Communication errors were predicted to only occur during task step 9, where the pharmacist would be communicating with the patient regarding their medication. During the focus group, participants discussed the difference between each of the potential communication errors, and how these would manifest into consequences of the errors. For example, if there was an ‘I1’ error during the consultation process, this would mean that no consultation occurred, leading to a patient potentially being unclear on the correct dosage for their medicines. While this was an issue, the participants agreed that the dose printed on the label would provide enough instruction to take the medicine safely, even if important issues were not mentioned (e.g., no leafy green foods whilst taking warfarin). This was similar to ‘I3’ error, where not all the information would be communicated; similarly, while this would be an issue if an important counselling point was missed, overall, the label on the medicine were considered to be sufficient. However, an ‘I2’ error was considered the most critical. This predicted that the pharmacist, or pharmacy staff, would communicate incorrect information to the patient. This would indicate a discrepancy between what would be on the label and what the pharmacist or staff member had said. This potentially could mean that the patient incorrectly takes the medicine, resulting in a sub-optimal therapeutic dose, or an overdose; or at the very least the patient would be confused about what dose they should be taking and require further consultation.

### 3.3. Probability, criticality and recovery

The majority of the potential errors were considered to have a low probability of occurring (n = 67, 76%) and low criticality if they did occur (n = 51, 58%). Only three sub-tasks (3%) were categorised as high criticality, with a further 6 (7%) categorised as both medium/high risk. It was identified through the SHERPA analysis that the majority of the errors, especially those of high criticality, can be recovered at task step 5. However, with the exception of step 5.2 any potential errors linked to step 5, if they were to occur, have no recovery step before the error reached the patient. This highlights the importance of step 5.2 in relation to the other steps, and the importance of the role played by the pharmacist who would exclusively complete this sub-task in practice.

### 3.4. Remedial measures

The suggested solutions ranged from training developments for individuals completing tasks, to work system changes and the incorporation of technology in specific sub-tasks. Once categorised as per the action hierarchy, it was identified that only 5 remedial measures were categorised as strong actions, with a further 14 intermediate and a further 16 as weak. All remedial measures suggested can be found in Table 4, categorised based on the action hierarchy.

**Table 4:** All remedial measures proposed with their corresponding action category, per the action hierarchy.

	Action Category	Suggestions	No.
Stronger Actions	Architectural/physical plant changes	• Separate areas for working on prescriptions to be processed immediately and regular medicines to be processed according to schedule	1
	New devices with usability testing	• Ensure ergonomic issues are appreciated (e.g., font and size of labels, utilisation of Tall-Man lettering)	1
	Engineering control (forcing function)		
	Simplify process	• Clear up no longer useful patient alerts, and ensure additional information inputted in the correct area • Ensure each patient has only one patient profile on the patient medication record system	2
	Standardise on equipment or process	• Introduce system to force staff to decide whether a patient is waiting for medicines instore, or returning (e.g., different colour coded baskets to process prescriptions)	1
	Tangible involvement by leadership		0
	Total Strong Actions		5
Intermediate Actions	Redundancy/back-up systems		
	Increase in staffing/decrease in workload	• Clear roles and responsibilities for all staff members • Ensure regular breaks for pharmacist and staff and mix tasks to allow for task switching when unfocused	2
	Software enhancements/ modifications	• Amend payment system to alert staff if incorrect amount of prescriptions charges processed • Utilise medicine bar codes for electronic check of medicines	2
	Eliminate/reduce distractions	• Reduce distractions and interruptions on pharmacist, and staff, when dispensing and checking medicines	1
	Checklist/cognitive aids		
	Eliminate look- and sound-alikes		
	Enhanced communication	• Include middle names in patient medication record profiles • Ensure all patient details are included in patient medication record profiles • Communicate medicine strengths on prescriptions in same format as supplied on the medicine • Provide prescriptions with similar amounts as pack sizes available (to avoid the need to dispense medicines in different boxes to the original) • Introduce standardised language to be used on prescriptions (including dosages)	5
	Simulation training with refresher	• Introduce meetings to reflect and learn on errors regularly occurring	1
	Review/enhancement of policy/guideline/documentation/workflow	• Include generic and brand name of medicines on prescriptions (where applicable)	1
	Review/re-evaluate use/appropriateness of equipment	• Ensure colour contrast is acknowledged with equipment • Ensure reference material are accessible on site and available during checking prescriptions	2
	Audit undertaken		
	Enhanced supervision		
	Implement a new team (frontline)		
	Standardised communication tools		
	Total		14
Weaker Actions	Double checks	• Ensure staff members sign medicines dispensed to ensure accountability	1
	Warnings and labels	• Provide reminders near medicines that are regularly dispensed incorrectly (e.g., stickers)	1
	New procedure/ memorandum/policy	• Marking prescriptions with initials of individual processing the prescription • Marking prescriptions with the total number of prescriptions to be processed for each patient (if more than one) • Comprehensive standardised procedure for processing prescription • Introduce cross checking of patient information once accessed patient medication record on the system • Ensure printing equipment properly maintained and serviced to ensure quality is acceptable and printing is clear • Separate high-risk drugs to allow for more focused concentration when dispensing	6
	Training and education (including counselling)	• Training on undertaking prescription checks • Training on exceptional medicinal products that require additional charges on a prescription • Trainings on high-risk medicines, and medicines with high potential of dispensing incorrectly (e.g., Look alike sound alike medicines) • Training on medicines with uncommon dosing regimens (e.g., Risedronate—once weekly) • Encourage good habits between team members to avoid negative learning from peers • Ensure suitably qualified individuals hand out medicines to allow for consultation • Introduce notice or leaflet to educate patients on importance of marking payment exemptions on prescription • Introduce sign regarding waiting times explaining the reason clinical and accuracy checks are necessary for the benefit of patient safety	8
	Additional study/analysis		
	Total		16

## 4. Discussion

This study has applied the SHERPA prospective risk analysis method to the process of dispensing of medication in community pharmacies for the first time. Our findings report on a broad range of potential remedial solutions, to 88 identified potential errors in the task of dispensing medicines in a community pharmacy setting. Task steps containing the highest potential for error have been highlighted, namely: ‘produce label for prescription’ and ‘complete final check for medicines’ and the most common type of error identified in the dispensing task was omitting a check. Our analysis therefore identified critical sub-tasks that if missed could lead to potentially serious errors, and has generated remedial solutions to avoid these errors occurring [27].

This study builds on the work conducted by Stojković et al. [19, 20], by providing more insight into the consequences that some of the errors and failures that may occur within the dispensing process. Additionally, by applying a systematic pre-set error taxonomy, more potential errors have been identified than in the earlier studies applying FMEA, however, it is not clear whether these additional potential errors are of the same significance as those identified previously or not. This is due to the variety of work and tasks completed within healthcare, and the difficulty with ascribing criticality values to the potential errors [28], as discussed below in the next section.

### 4.1. Error likelihood and criticality

One difficulty when completing the SHERPA analysis was deciding on the likelihood and criticality values. With regards to the likelihood, each participant had their own opinion and own experiences, which ultimately led to differing suggestions on the likelihood of various errors occurring. For example, when discussing errors related to the sub-task analysing the dispensing of medicines into baskets, participants mentioned that staff training and experience had a large effect on the likelihood of this error occurring, and similarly their previous experience with medication errors usually ensured they were more careful with medicines with a high potential of error occurring. This meant that each pharmacist had a slightly different experience with some of these errors, which is consistent with what has been reported in general with regards to these methods within healthcare [28]. Regarding the criticality of the error, the participants discussed the difficulty with deciding on a “score” due to the wide effects that could occur depending on the context. For example, with regards to a dosing error, if the instructions on a paracetamol prescription were incorrectly labelled, “two tablets four times a day” instead of “one tablet four times a day” this would be ranked low, however, if that patient had an underlying problem with their liver, or if the dose was printed as “three tablets” instead of “two”, each of these differences would immediately be considered a high rank of criticality. This means that both these ratings should be taken as more representative of the average likelihood and criticality and would be subject to change depending on the specific case and cannot be taken as definitive or comprehensive of all predicted errors in this analysis. This issue was also highlighted by other SHERPA analyses completed [25], that while a SHERPA provides a thorough and systematic list of errors, task-specific details would alter the errors, and more specifically their likelihood of occurring and the criticality of the error if it occurs. One perspective that has been suggested with regards to the validity of the likelihood and criticality scores is to use consensus scoring, as we have in this study, rather than a mathematical procedure [29]. Another suggestion has been to combine data from incident databases to improve the reliability of these ratings [30, 31] and improve validity of the findings [32], but the main focus of these methods has been on the qualitative insights they provide [30, 31].

### 4.2. Recovering from errors

It was highlighted through the SHERPA analysis the importance of task step 5, which was the final, and sometimes only, opportunity to recover from many errors completed prior in the task. This step is exclusively completed by the community pharmacist, and points to the pressure that is faced by the pharmacist in ensuring no errors occur during their final checks, and the reliance of this risk control on the ability of the human operator to detect a discrepancy. This becomes critical when viewing human error from a HFE perspective which is, that it is unachievable to remove human error from a system, and the system must adapt to minimise the likelihood of an error occurring. In line with HFE principles, greater focus should be applied to either implement more risk controls to prevent the occurrence of the error (i.e. error reduction), such as reducing distractions during checking, or implementing more risk controls to improve detection and recovery from errors (i.e. resilience), for example leveraging bar code scanning to ensure the correct medicines are dispensed [33, 34]. One potential solution suggested to reduce the risk with this single sub-task is separating the different checks, and conducting a clinical check (i.e., ensuring the medicine is suitable for patient and their condition) prior to the dispensing, and only completing an accuracy check (i.e., ensuring medicine is dispensed correctly based on the patient’s prescription) once the medicine has been dispensed and labelled. Additionally, it was also suggested that the importance of work breaks is reiterated to ensure concentration when completing these sub-tasks, as well as introducing policies to reduce interruptions and distractions during this sub-task.

### 4.3. Remedial solutions

The objective of completing any error prediction method is, ultimately, to provide solutions for the errors identified. It is noteworthy to recognise that not all potential solutions are viewed equally effective from an HFE perspective. HFE is a system and design-based discipline [35] and so, from an HFE standpoint, remedial measures should specifically target the redesign of the system to avoid any potential errors, rather than focus on behavioural interventions to the human operators in the task. This is the premise upon which the US Veteran Affairs National Centre for Patient Safety action hierarchy is based.

The remedial measures proposed by the pharmacists during the focus groups were categorised based on the action categories identified by the action hierarchy. Out of 35 remedial measures proposed by the focus group participants 16 (46%) and 14 (40%) were categorised as weak and intermediate, respectively. Only 14% of measures proposed were considered strong. This shows a preference from the participants to suggest interventions that primarily focused on adapting and manipulating the behaviours of individuals completing the tasks. While the dispensing task may not require stronger interventions, an alternative explanation could be the limited training that pharmacists receive in HFE principles during their training, or there are resource constraints that would limit the ability to redesign the system. Studies have identified that within the UK, HFE principles are not covered enough within undergraduate pharmacy curriculums [36], and further studies investigating HFE within community pharmacies have recognised that not enough guidance is available to support pharmacists in applying HFE principles within their practice [37].

### 4.4. Study strengths and limitations

While a SHERPA analysis can be conducted by a single individual, a strength of this study was the focus group discussions with experienced community pharmacists, which provided multiple insights as well as creative remedial measures through the discussions. However, all participants conducted the same role (i.e., community pharmacists), with similar experiences which may lead to limited range of ideas, with a limited sample size of pharmacists participating Future work could apply this same method but utilise a wider range of participants and stakeholders involved with community pharmacy practice (e.g., pharmacy leaders, regulators, HFE experts), to elicit more fundamental changes to tackle the errors highlighted in this study. For example, while utilising bar code scanning was mentioned as a potential remedial measure, other measures that may require significant financial investments (e.g., introducing robotic dispensing) were not mentioned, even though they may solve a similar problem through a similar approach (i.e., introducing technology into the task). While participants may have not believed that this was a suitable remedial measure, another explanation could be that participants were limited to measures they believed were in their control, missing other measures that would require more senior leadership stakeholder involvement, or financial resources beyond their means. Additionally, the SHERPA analysis focuses on proposing measures aimed at tackling potential errors specific to individual sub-tasks, which may fail to identify holistic system changes and interventions that could reduce the burden of errors within healthcare [38].

In addition to this limitation of the SHERPA analysis, there are other limitations that should be considered, especially if SHERPA is to be utilised within practice. Due to SHERPAs prospective outlook, it can be particularly useful in not applying blame when discussing risk and potential errors, however this process can become laborious and time-consuming, as the use of a SHERPA analysis is determinant on the availability of an HTA. This can result in the complete process requiring significant investment from a time and effort perspective. Additionally, the use of an HTA presents the task in a linear process, with tasks being completed from the first sub-task to the last according to the plan in order to achieve the goal. However, in the reality of healthcare, and community pharmacy in particular, pharmacists may be required to manage multiple tasks or rearrange some sub-tasks based on resource availability and thus the HTA should not be considered as the only way for the dispensing task to be completed, and with these interactions further risks and potential errors may be introduced.

### 4.5. Implications for policy and practice

This SHERPA analysis has identified the most critical parts of the dispensing task, as well as shining a light on the importance of the pharmacist role during dispensing, as a last barrier before medication errors reach the patient. In an attempt to mitigate the potential errors highlighted in this study, remedial measures have been proposed by experienced pharmacists. It is important however, that any proposed remedial measures are assessed first before wide-scale implementation, to confirm that they are indeed effective and will contribute to improvements in patient safety. This study used the US Veteran Affairs National Centre for Patient Safety action hierarchy to categorise the proposed remedial measures based on their reliance on individual human behavioural change for improvement, with solutions tackling system changes and architectural improvements to reduce potential errors. The majority of remedial measures identified were categorised as weak or intermediate, with only 5 (14%) of measures proposed categorised as strong interventions. While the dispensing task may not require strong remedial measures, engaging a wider stakeholder base (e.g., Human Factor experts, senior managers at pharmacies) may uncover other wider-reaching interventions, based on the seniority or knowledge of the participants.

Additionally, researchers within the pharmacy practice domain have already begun investigating the reallocation of tasks from the pharmacist to other pharmacy staff within a community pharmacy setting [39, 40]. These studies have identified the utilisation of the team around the pharmacist as a key initiative to provide the pharmacist with the time to conduct alternative clinical tasks, for the benefit of the patient. Future work can utilise the findings of this study to ensure that risks and potential errors stemming from tasks investigated in this study and under consideration for reallocation are not introduced, as well as explore the potential of introducing technology or machines to reduce the potential of human error in these sub-tasks.

## 5. Conclusion

This study has applied an established HFE method to identify potential errors within dispensing in community pharmacy practice. Altogether, 88 potential errors were identified, and 36 remedial measures suggested to prevent these errors from occurring. The remedial measures were evaluated based on an established patient safety action hierarchy, which categorises errors as strong, intermediate, or weak based on their remit and likelihood at succeeding, according to HFE principles. Future work should evaluate and potentially pilot the proposed remedial measures to identify whether they lead to improvements in the management of risks in the dispensing task. Additionally, engaging a wider stakeholder base may identify whether wider-reaching remedial measures exist, to support those identified in this study.
